# Bird Community Colours Across Different Types of Habitat

**DOI:** 10.3390/ani16050815

**Published:** 2026-03-05

**Authors:** Federico Morelli, Yiming Deng, Paolo De Fioravante, Andrea Strollo, Riccardo Santolini, Paolo Perna, Yanina Benedetti

**Affiliations:** 1 Department of Life Sciences and Systems Biology, University of Turin, Via Accademia Albertina 13, 10123 Turin, Italy; 2 Faculty of Environmental Sciences, Czech University of Life Sciences Prague, Kamýcká 129, 16500 Prague, Czech Republic; dengy@fzp.czu.cz (Y.D.); ybenedetti73@gmail.com (Y.B.); 3 Italian National Institute for Environmental Protection and Research (ISPRA), Via V. Brancati 48, 00144 Rome, Italy; paolodefioravante@gmail.com (P.D.F.); andrea.strollo@isprambiente.it (A.S.); 4 Department of Humanities, Università Degli Studi di Urbino Carlo Bo, 61029 Urbino, Italy; riccardo.santolini@uniurb.it; 5 Terre.it Srl, largo Decio Filipponi n◦30/a, Palazzo Costa, 62028 Sarnano, Italy; paolo.perna@terresrl.it

**Keywords:** avian community, colouration, habitat type, forest, grassland

## Abstract

(1) The bird colouration is the result of adaptation to environmental conditions, predator–prey relationships, and sexual selection (intraspecific competition and signalling of quality). Only a few studies have explicitly explored the plumage colouration of birds at the level of species communities. (2) Methods: We combined data with bird plumage colours and their spatial distribution at a large spatial scale in Italy, exploring the relationship between community colours and different types of habitats and landscape heterogeneity. (3) Results: Overall, we found that the more representative colours of avian communities were grey, white, black, and brown. The percentage of black colour in the community was smaller in close habitats (e.g., forests). A high percentage of brown was observed in forests and shrublands, whereas a high percentage of white was found in wetlands, water bodies, and urban areas. The percentage of yellow was relatively low overall, but it was slightly higher in deciduous forests. Land use richness increased the percentage of brown, green, rufous, and yellow, while negatively affecting other pigments (black and grey = melanins, purple = structural, and red = carotenes). The community colour inequality decreased when the species and land use richness increased, while it increased when the weighted edge density of surrounding landscapes increased. Finally, we found that bird communities that are made up of closely related species show a wider variety of colours (e.g., lower colour inequality). This supports the idea that closely related species that live together develop different features to improve species recognition. (4) Conclusions: We found that the colours of bird communities are related to the type of environment.

## Simple Summary

Bird colouration influences interactions with the environment and between species. However, studies focusing on the colouration of bird communities are scarce. This research utilized a large dataset to examine the relationship between community colours and different habitats in Italy. The dominant colours were found to be grey, white, black, and brown. Black was less common in closed habitats like forests, brown was prevalent in forests and shrublands, and white was more common in wetlands, water bodies, and urban areas. Yellow was generally rare but slightly more frequent in deciduous forests. Increased landscape heterogeneity led to a rise in brown, green, rufous, and yellow percentages but decreased melanins and structural colours. The overall colour inequality of bird communities decreased when increasing the number of species and land use richness, while it increased with higher edge density. Notably, communities with closely related species in the same habitat showed greater colour diversity, suggesting that they evolve distinct features for better species recognition.

## 1. Introduction

The colouration of birds is the result of adaptation to environmental conditions and predator–prey relationships, and it is subject to aspects of signalling in sexual selection (intraspecific competition and proxy of quality), as well as other biotic interspecific interactions [1]. There are three main types of production of the bird feathers’ colouration: from either pigments (e.g., carotenoids, melanins, and porphyrines), from light refraction caused by the structure of the feather (e.g., iridescent colours), or from the combination of both mechanisms [2,3,4]. Carotenoids are responsible for red, bright yellow, and olive green when combined with melanins [3,5,6]. Melanins are responsible for the darkest blacks, rose-coloured browns, and pale yellows [7]. Finally, porphyrins are responsible for the colours pink, brown, red, and green [8,9]. On the other hand, the colours produced by the structure of the feather are responsible for iridescent colours and the non-iridescent colour blue [10,11].

The remarkable variation in bird colouration (phenotype) among species is associated with their genotype [12] and is primarily attributed to sexual selection, particularly in polygynous mating systems [13,14]. Thus, more colourful or more elaborately coloured males are positively selected by females, while natural selection favours less elaborate colours in females [13]. For example, black, red, and blue colours occur more often in males, while females tend to be more cryptically coloured in the case of bird species where the eggs are primarily incubated by the female [13,15]. Males also tend to be darker coloured than females [16]. Also, social selection can influence a bird’s colouration: certain colours make males more likely to retain their territories and win contests compared to others [17], as well as being associated with male–male competition when colours are used to signify dominance and status [15,18]. However, the mechanisms behind the colour evolution in birds are numerous and complex, and determining the relative importance of various explanations for birds’ colour diversity has proven challenging [3,19].

Some colours are primarily associated with the species’ diet [20]. A high-protein diet can produce more colourful crown feathers in birds, increasing iridescent ornamental feathers [21]. Carotenoid-derived colours are among the most commonly used in birds, and there is a strong association between carotenoid availability and food availability in their plumage colouration [22,23,24]. Colours are also associated with body size. Larger bird species (especially in some groups, such as the Psittaciformes) tend to be darker and exhibit blue or red plumage colours more often than smaller species [25]. Overall, small bird species tend to be characterized by lighter shades such as yellow, light grey, green, light brown, dark brown, and white [15]. Yellow and red colours, pigments primarily based on carotenoids, tend to be more common in smaller species [26]. Body size also affects plumage colour heterogeneity in birds, and there is a negative relationship [26] between the two. The birds’ colouration is linked to the level of conspicuousness or elusiveness of the species. The balance between conspicuous and mimetic or hidden colours is associated with the cost of predation risk and the anti-predator strategies of birds [20]. More coloured birds are overall more easily detected in the natural habitats and consequently more exposed to predation risks [27,28], except for aposematism (i.e., when conspicuous colours are used as warning colouration or anti-predator adaptation) [29].

The birds’ colourfulness is also linked to a latitudinal gradient in passerines towards the equator [30,31]. The strong latitudinal increase in passerine birds’ colourfulness towards the peak of the tropical zone is partially explained by latitude-associated gradients in climatic conditions [30]. The variation in plumage colouration of birds is linked to climate variables, following Gloger’s rule, with achromatic variations associated with variables such as temperature and precipitation [32,33]. Research indicates that the amount of annual rainfall is positively correlated with darker plumage colouration in several species [34]. This suggests that darker morphs in humid habitats may provide better camouflage against the darker habitat and/or repel water more effectively [34]. Finally, a negative correlation has been observed between melanin pigmentation and temperature in various species, suggesting that darker animals tend to be more prevalent in colder environments, likely for thermoregulation purposes [32,35]. This indicates that dark pigmentation may restrict the geographical distribution of various bird species, due to the thermal niche limitations [36].

One of the most important factors influencing the evolution of bird colours is the habitat type in which these species reside [15,37]. A significant association was found between birds’ plumage colouration and their habitat use [38]. The intensity of light in various habitats influences the evolution of interspecific variation in plumage colours [39]. It was found that more conspicuous colours are often associated with closed habitats, such as forests, rather than with open ones [26,40,41]. On the one hand, darker-coloured species (e.g., birds with a high percentage of black) tend to be more indicative of habitats with dense vegetation, such as forests [37,38,42]. Forest habitats can also slightly reduce birds’ overall chromaticity, potentially increasing their crypsis or mimetism [37,43]. On the other hand, species with white patches, which increase their conspicuity, can be more commonly found in closed habitats, such as forests [44]. Conspicuous colours may be more common in closed habitats than in open habitats, as they are associated with a relatively lower predation risk in such habitats [26,40]. The birds’ plumage colours are part of an urban-associated syndrome, potentially driving an overall homogenization of the colouration [45,46,47]. Additionally, urban habitats can filter out megacolorful species by reducing the niche suitability for most diet-specialized and smaller-bodied passerines [30,48]. The colour homogenization associated with urbanization can reflect the biotic homogenization of avian communities, a phenomenon well documented in recent years in several European cities [49,50,51]. Biotic homogenization could also alter the colour composition of bird communities because species that are more closely related phylogenetically often show similarities in both appearance and behaviour [52]. However, when these species coexist in the same geographic region, their appearances may diverge to enhance species recognition [53,54].

Until now, only a few studies have explicitly explored the plumage colouration of birds at the level of species communities [30,37,40,45,50,55]. Avian communities play many roles in nature, contributing to various ecological functions and ecosystem services. Forest bird communities differ from those in open habitats, grasslands, or farmlands in terms of species richness, as well as their functional traits [56,57,58] and their potential use as bioindicators [59]. Avian community composition is shaped by a combination of local and landscape factors, such as vegetation cover and characteristics [60,61], abundance of resources (e.g., food, nesting sites, etc.) [62,63,64], surrounding landscape type and heterogeneity [65,66], and different types of interspecific interactions (e.g., predation, competition, etc.) [67,68].

Our understanding of the potential link between the dominant colours of avian communities and their preferred habitats remains incomplete. Furthermore, other environmental characteristics, such as landscape heterogeneity, can influence the relative composition of birds of different colours within the species communities. There is a substantial amount of research investigating the relationship between the presence of different bird species, particularly in terms of increased species richness, and the amount of marginal or residual vegetation in landscapes, especially in rural areas [69,70,71,72,73]. We expect that increasing landscape configurational heterogeneity, such as edge density [74,75], can change the composition of avian communities by increasing species richness [76], potentially altering the balance of colouration within the community.

In this study, we combined a large published dataset with information on birds’ plumage colours [15] and species’ spatial distribution across Italy. We calculated the relative colour composition of bird communities and explored which dominant colours were associated with different habitats. We then tested whether the overall evenness of colouration across communities (i.e., colour inequality) was influenced by landscape heterogeneity or by characteristics of bird communities, such as species richness and phylogenetic relatedness. Phylogenetic relatedness describes the level of phylogenetic association among species in the community [77] and can offer additional and complementary information on biodiversity in terms of evolutionary heritage [78].

We have three main expectations regarding the relationship between plumage colours and environmental characteristics [37,38,46]:

(A) We expect that each habitat type will feature a distinct colour composition in bird communities.
(B) We expect to find that communities dominated by specific colours can be associated with particular habitat types, such as darker-coloured communities in forests and lighter-coloured communities in open areas like arable land and grasslands [44].
(C) We expect that landscape heterogeneity will significantly impact the relative composition of plumage colours, potentially reducing colour inequality or dominance within bird communities. This expectation is based on the edge effect, which refers to changes in population or community structure that occur at the boundaries between two or more habitats. This phenomenon is well-documented in ecological studies and may attract a higher number of species [79,80].

Finally, we expect that communities with species belonging to fewer lineages (e.g., more phylogenetically related) may be characterized by increased colour diversity (e.g., the predominance of many different plumage colourations) to enhance the species recognition signals, potentially reducing the risk of hybridization [53,81].

## 2. Materials and Methods

### 2.1. Bird Species Distribution, Type of Habitat, and Species Colouration

Data on bird species distribution were obtained from a national project in Italy, developed by Lipu and BirdLife Italy, and funded by the Ministry of Agriculture, Food and Forestry. The Italian project mentioned above focuses on the composition of breeding bird communities [82]. This project involves a standardized monitoring scheme, led by experienced ornithologists and volunteers across the country, to count breeding birds at randomly selected sites each spring. Point counts of 10 min with an unlimited distance [83] and a single visit [84] were used during the years 2018, 2019, and 2020. All individuals observed or heard during the 10 min observations were recorded. A more detailed explanation of the survey procedure is available at https://www.reterurale.it/farmlandbirdindex (accessed on 26 February 2026). We combined all breeding bird species present during the three years of the survey to comprehensively characterize each avian community and reduce potential bias from the differences in species detectability [85]. In this study, we defined a species as present if it was recorded at least once over a three-year period of monitoring. If a species had never been recorded, we classified it as not present. Our goal was not to explore changes in bird communities over time but to identify the most representative species in different habitats. Thus, we created a matrix indicating whether each bird species was present or absent, as well as the total number of species (species richness) at each sampling site. In each bird community, we calculated the degree of shared evolutionary history by estimating the metric ‘phylogenetic relatedness’ (PSVs) [86,87]. To calculate PSVs, we first randomly downloaded 100 species-level phylogenies (using the “Hackett backbone”) from the BirdTree web tool (http://birdtree.org) [88]. Then, we constructed the maximum clade credibility tree (MCC) using these phylogenies and using the function ‘maxCladeCred’ in the library ‘phangorn’ v. 2.8.1 package [89]. PSVs were estimated using the ‘Picante’ ver. 1.7 package for R [90].

To describe the environmental conditions surrounding each point count, we created a buffer area of a 200 m radius as suggested in a previous study [91]. We overlapped such buffers with the Italian 10 m resolution land use map that was derived from the integration of the national land consumption map with the main land cover/land use data of the Copernicus land monitoring service [92]. The dominant habitat type of each buffer (hereafter referred to as the “avian community”) was classified based on the higher percentage of different land uses. The land use types considered in this study are the following eleven categories: coniferous forest, deciduous forest, shrubs, non-agricultural herbaceous vegetation, wetlands/water, bare soil and dunes, orchards and vineyards, forage, croplands, urban areas, and mixed habitats. The percentage of each land use type was calculated using the “intersect operator” function in ArcGIS 10.8.1 [93]. Each 200 m radius buffer was classified as the dominant habitat when the main land use cover was >60% [91], except for the category urban, which was classified as dominant when such land use type occupied ≥30% and all other categories individually comprised ≤ 60% of the land area. Buffer areas with relatively mixed composition, where none of the land use types occupied at least 60% of the area, were classified as mixed habitats [91]. Buffer areas with an overall forest coverage ≥60% were subdivided into the deciduous forest category when deciduous forests occupied ≥40% and coniferous forests occupied ≤20%, and were subdivided into the coniferous forest category when coniferous forests occupied ≥40% and deciduous forests ≤20%. Based on the relative composition of land use in each buffer area, we estimated two proxies of the landscape heterogeneity: (1) land use richness (LUR), which was estimated simply as the total umber of different types of land uses within the 200 m radius buffer, and (2) weighted edge density (WEDGE), which was calculated as the sum of the perimeters of all polygons in the buffer area multiplied by the number of land use richness (LUR) [94]. Both landscape metrics have been previously suggested as potential proxies for changes in avian species composition [95,96].

In this study, we utilized the colouration of each bird species that was extracted from a recent study [15]. Delhey et al. (2023) used bird plates (e.g., ‘png’ files) from the *Handbook of the Birds of the World* [97] to extract the primary human-visible CIELAB colours that characterize the plumage and some soft parts such as the bill, legs, and eyes. The main colours were classified into different variable colours, computing the proportion of the pixels in each image that fall within specific sections of colour space [15]. In our study, we reduced the number of variable colours by grouping light and dark grey and light and dark brown, respectively, into “grey” and “brown”, obtaining the 10 most common colours (e.g., black, blue, brown, green, grey, purple, red, rufous, white and yellow) that were used in further analyses. Additionally, we only filtered male individuals for all species, considering that males are more colourful than females [98]—a pattern repeated in almost all Italian bird species—making males more likely to highlight large spatial patterns at a community level. For each bird species, we compiled information regarding the main colours characterizing the plumage and some soft parts, expressing them as a percentage of 10 variable colours. Colours that were more representative of the species (e.g., colours with a greater composition) achieved the highest values in %.

We combined the dataset on bird species colouration with the dataset on bird species presence or absence in each sampling site to obtain the relative composition of colouration in each avian community. To do this, we first summed the colour of each species present in the community and then recalculated the relative composition of each colour (Figure S1). We calculated the relative colour percentage in each avian community and expressed them as a value (%) between 0 and 100 (e.g., black percentage = black sum ∗ 100/sum of all colours in the community). Finally, we calculated the colour inequality in the bird communities using the Gini coefficient [99]. We calculated the Gini coefficient on the ten variables with the relative composition by colours (e.g., colour percentage), using the library ‘DescTools’ for R [100]. The Gini coefficient is a measure of statistical dispersion that ranges between 0 and 1 and represents low to high inequality. In the specific case of community colours, a value near zero indicates the highest level of colour uniformity in the community (e.g., each colour is equally represented). In contrast, a Gini coefficient of 1 indicates the highest inequality level, for example, when a single colour characterizes the whole avian community.

### 2.2. Statistical Analyses

We explored the changes in the percentage of each different colour (e.g., black, blue, brown, green, grey, purple, red, rufous, white and yellow) and colour inequality in the breeding avian communities, concerning the type of habitat, configurational and compositional landscape heterogeneity, the number of bird species in the community and latitude and longitude of the sampling sites. We ran a generalized linear model [101] for each different colour (e.g., response variables). The following variables were introduced as predictors: dominant habitat, land use richness (LUR), weighted edge density (WEDGE), bird species richness, longitude, and latitude. We incorporated the longitude and latitude into the modelling procedure to alleviate any potential spatial autocorrelation (SAC) in the data. Spatial autocorrelation was tested for each response variable using Mantel tests (Guillot, 2011), with 999 Monte Carlo permutations [102] (Table S1). The Mantel statistics range between −1 and 1, like a correlation coefficient [103], and evaluate the congruence between two matrices (one matrix measuring the distance among variable values and the other one measuring the geometric distance among geo coordinates of sampling sites [104]). Because we found weak but statistically significant spatial autocorrelation in several response variables that we focused on (Table S1), we decided to incorporate latitude and longitude as predictor variables in the further modelling procedure. We also run a generalized linear model (GLM) for colour inequality as the response variable and dominant habitat, land use richness (LUR), weighted edge density (WEDGE), bird species richness, phylogenetic relatedness (PSVs), longitude, and latitude modelled as predictors. Additionally, we run a generalized linear mixed model (GLMM) [105] with colour inequality as the response variable and land use richness (LUR), weighted edge density (WEDGE), bird species richness, phylogenetic relatedness (PSVs), longitude, and latitude modelled as predictors, while dominant habitat was incorporated as a random factor. The GLMM aimed to verify whether the associations identified in the initial model regarding colour inequality remain significant when considering potential habitat-related differences. The model’s overall fit was evaluated using the pseudo R-squared value for GLMM [106].

All statistical analyses and data explorations were performed in R software v. 4.1.1 [107].

## 3. Results

**Figure 1:**
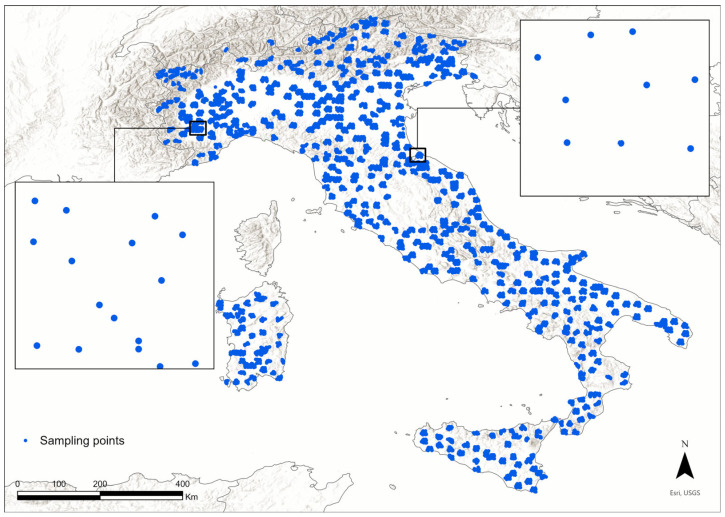
The study area covered the entire country of Italy, with the geographic positions of the sampling points where data on the composition of breeding avian communities were collected. The zoom areas (e.g., the small boxes) illustrate the spatial distribution of the sampling sites, with a minimum distance of 200 m between sites to mitigate the risk of double-counting.

**Figure 2:**
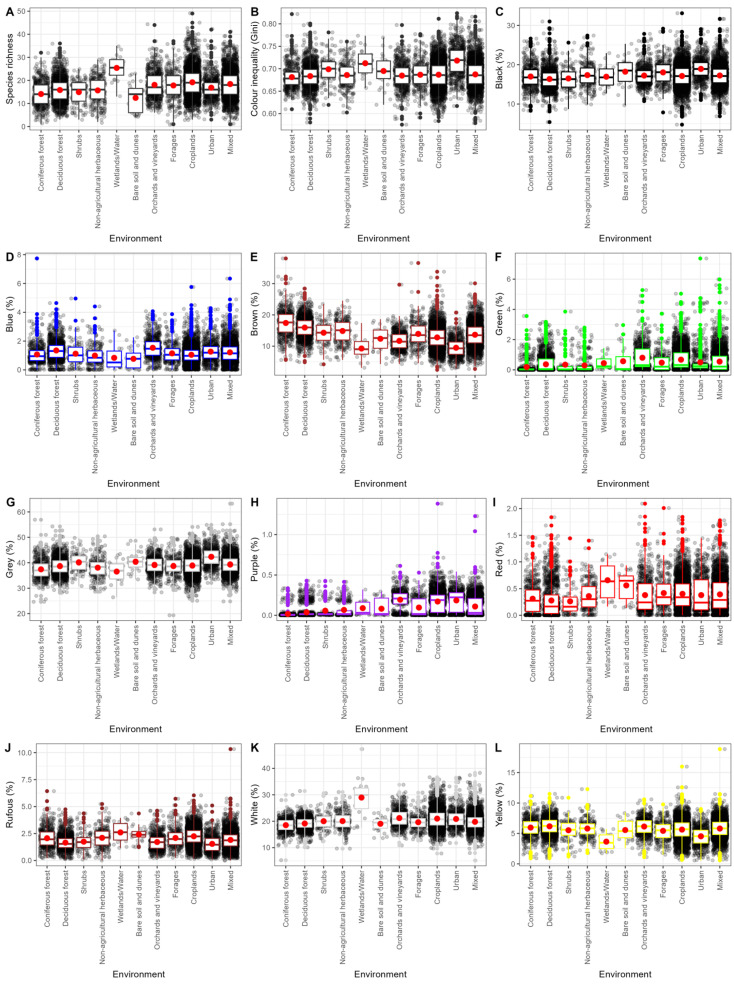
Species richness (**A**), the colouration diversity or inequality (e.g., Gini coefficient) (**B**), and the percentage of different colours (e.g., black (**C**), blue (**D**), brown (**E**), green (**F**), grey (**G**), purple (**H**), red (**I**), rufous (**J**), white (**K**) and yellow (**L**) in the breeding avian communities by type of habitat. Box plots show the median (the coloured bar in the middle of the boxes); upper and lower quartiles (length of boxes); the maximum and minimum values (whiskers); and the mean values (red dots). N = 8508.

A total of 262 bird species were recorded at 8508 sampling sites during the breeding season in Italy (Table S2, Figure 1 and Figure S2), exhibiting considerable variation in species richness across different habitat types. Habitats with higher bird species richness included riparian areas, such as wetlands and water bodies, followed by croplands and mixed habitats. In contrast, a lower number of bird species was recorded in bare soil and dune areas, as well as conifer forests (Table 1, Figure 2A). The avian communities with more closely related species (a lower value of PSVs) were from coniferous forests, while the less closely related species were found in wetlands and water bodies, followed by communities from croplands (Table 1).

Overall, the most dominant colours were grey (39.1%), white (20.2%), and black (17.2%) (Table 1). The breeding communities of birds in different habitats were characterized by distinct colour inequality (Table 1, Figure 2B) and colour compositions (Table 1, Figure 2C–L).

The higher mean inequality was observed in urban, wetland, and water areas (e.g., higher dominance of a single colouration) (Figure 2B, Table S4), while the maximum inequality was observed in urban areas (Table S4). In contrast, a relatively higher uniformity in colour composition (e.g., a minimum inequality) was found in orchard vineyards (Figure 2B, Table S4). These results were also highlighted by the generalized linear model outputs (Table 2 and Table S5).

**Figure 3:**
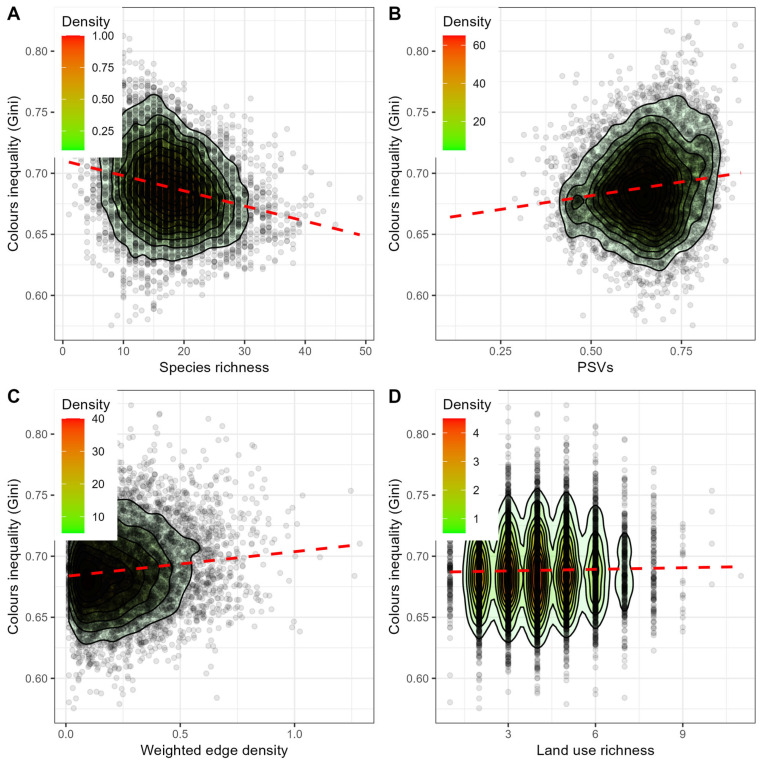
Colour inequality in the breeding avian communities (e.g., Gini coefficient) vs. species richness (**A**), phylogenetic relatedness (PSVS) (**B**), weighted edge density (**C**), and land use richness (**D**). The points are distributed according to the density function, and the red dotted lines indicate the linear regression between each pair of variables.

Moreover, colour inequality was negatively correlated with total species richness and land use richness (Table 2, Figure 3A and Figure 3D, respectively), while it was positively correlated with phylogenetic relatedness (PSVs) and weighted edge density (Table 2, Figure 3B and Figure 3C, respectively). The same significant associations between colour inequality and species richness, phylogenetic relatedness, land use richness, and weighted edge density were confirmed by the generalized linear mixed model outputs (Table S6).

The results of the multi-model procedure indicated that the percentage of black in avian communities was negatively associated with forest cover, while it increased with latitude (Table 2). The percentage of blue was positively associated with almost all habitat types except non-agricultural herbaceous, wetlands/water, and bare soil and dunes, while it decreased with latitude (Table 2). The brown percentage was lower in wetlands, water bodies, and urban areas, increasing with latitude (Table 2). Green was negatively associated with coniferous and deciduous forests, shrubs, non-agricultural herbaceous vegetation, and wetlands/water (Table 2) and with latitude. Grey was negatively associated with almost all habitat types except shrubs, bare soil, dunes, and urban areas, and it decreased with both latitude and longitude (Table 2). Purple was slightly higher in orchards and vineyards, as well as in cropland and urban areas, and it increased with latitude (Table 2). Red colour was negatively associated with almost all habitat types except wetlands/water and bare soil and dunes, while it increased with latitude (Table 2). Rufous colours increased with latitude, while they were negatively associated with almost all types of habitats except non-agricultural herbaceous, wetlands/water, bare soil, dunes, and croplands (Table 2). A relatively higher percentage of white was found in wetlands and water, as well as in orchards, vineyards, croplands, and urban areas, and it decreased overall with latitude (Table 2). The yellow colour was only significantly positively associated with deciduous forests and was unrelated to latitude, but it was positively associated with longitude (Table 2).

Landscape heterogeneity had contrasting effects on the relative colouration of avian communities. Land use richness increased the percentage of brown, green, rufous, and yellow, while negatively affecting all pigment types, including black and grey (melanins), purple (structural), and red (carotenes) (Table 2). Additionally, the weighted edge density was positively correlated with the percentages of black, blue, grey, purple, and red and was negatively correlated with the percentages of brown, green, rufous, and yellow (Table 2). Finally, we found contrasting (e.g., positive and negative) associations between the percentage of each colour and the total number of birds (Table 2). White was the only colour not related to species richness. Brown, green, red, rufous, and yellow increased with species richness, while black, blue, grey, and purple decreased with species richness (Table 2).

## 4. Discussion

In this study, we examined changes in the relative composition of colours in bird communities with respect to ecological attributes (e.g., habitat type and heterogeneity). The dominant colours of Italian bird communities were grey, white, black, and brown; however, their relative proportions varied significantly across habitat types and landscape characteristics. Darker colours, such as a higher percentage of black, were less common in the darkest habitats, like forests. At first glance, this finding appears to contradict previous studies that indicated darker-coloured species are more likely to inhabit forested areas [37,38]. However, forests may not only support the darkest-coloured species but also those with less elaborate and less vibrant colourations [43]. Additionally, white patches in the plumage of certain species can also be commonly found in enclosed habitats, such as forests [44], potentially changing the overall colour composition of the communities. Finally, these discrepancies could be linked to intrinsic differences in communities and the biogeographical areas in which the studies were conducted (e.g., Italian vs. Neotropical rainforest bird communities).

The presence of blue-coloured bird species in communities was observed at a relatively low percentage of less than 1.2%. This colouration was even less prominent in non-agricultural herbaceous areas, wetlands, water bodies, bare soil, and dunes. We speculate that Italian bird communities are less characterized by the presence of blue-coloured bird species than those found in tropical regions. Interestingly, blue-coloured birds in tropical rainforests tend to be more abundant in forests characterized by a greater canopy [108]. In our study, which focused on a large spatial scale, it was impossible to distinguish the forest areas by considering the different sections of the forest (e.g., canopy, subcanopy, and understory). We found a higher percentage of brown colour in forests and shrubland communities than in communities from other habitat types in Italy. The presence of brown species, commonly associated with the understory of forests, was already reported in a previous study focused on tropical rainforests and may be associated with the increased beneficial camouflage afforded by such colouration of the species [108]. We believe the same explanation applies to our communities in forests and shrublands.

Our results indicate that the colours green, grey, red, and rufous are primarily negatively correlated with certain habitat types, and no positive correlations were found. For example, the model outputs suggest that these colours are less common in forested areas. In contrast, the percentage of white colours in avian communities increases in specific habitats, such as wetlands and water areas, orchards and vineyards, croplands, and urban areas. We can try to identify some species responsible for these habitat-related changes in community colouration. In wetlands and water bodies, several waterbird species exhibit predominantly white plumage, including *Larus michahellis*, *Recurvirostra avosetta*, *Ardea ibis*, and *Egretta garzetta*. The presence of these commonly observed species (as shown in Table S2) contributes to the overall abundance of white colouration in these communities. The high percentage of white colour among waterbird communities in wetlands and other open habitats may offer a more conspicuous signal for species recognition. This has been suggested as a potential long-range signalling advantage in such habitats [38]. Additionally, the typical colouration of waterfowl (e.g., gulls, terns, skimmers, grebes, plovers, and ducks) is characterized by a light (white) lower half of their body and a dark upper (dorsal) part, which functions as a kind of camouflage against aquatic predators because light ventral surface renders them cryptic when viewed from below against the sky or downwelling light [109]. In urban areas, on the other hand, species such as *Motacilla alba*, *Pica pica*, *Larus michahellis*, *Delichon urbicum*, and *Aegithalos caudatus* can significantly contribute to the high percentage of white colour that is present in local bird populations. Urban bird communities can exhibit a prevalence of white colouration, as urban areas tend to experience higher temperatures compared to surrounding regions. This phenomenon is attributed to the urban heat island (UHI) effect, which has been well documented in various cities [110,111]. White—and other lighter colours—can offer a thermoregulatory advantage to some species in warmer environments, as they help birds stay cooler by reflecting more solar radiation [32,112]. In urban areas, we also found a positive correlation with blue and purple colours in the bird community, while a negative correlation with brown, rufous, red, and yellow colours. Our results are partially consistent with a previous study suggesting that blue, dark grey, and black are more typical species in urban areas, while brown and yellow are less typical [46]. The yellow colour is relatively uncommon in Italian avian communities, accounting for less than 5.8% of the total colouration observed, and we found it to be slightly more prevalent in deciduous forests. One possible explanation for this is the abundance of lepidopteran larvae, which are particularly common in deciduous forests and can serve as an important dietary component for several bird species [113]. Butterflies and their larvae provide a significant source of carotenoid pigments for many birds [114,115]. Among the yellow-coloured bird species found in these forests are *Oriolus oriolus* and smaller Passeriformes such as *Parus major*, *Cyanistes caeruleus*, and *Phylloscopus sibilatrix*. These species can help us explain the positive association between the yellow colour of carotenoids and deciduous forests. Forests (e.g., deciduous and coniferous) were characterized by lower colour inequality in avian communities, suggesting communities composed of species with plumage colouration that is more diverse than in other types of environments, such as wetlands or urban areas. We think this result could be associated with intrinsic differences in niches offered by the category forests (here heavily simplified into two main categories, for example, deciduous and coniferous). As explained in previous studies, dense vegetation, such as forests, can represent contrasting light environments, with a bright canopy and a dark understorey, where both areas potentially host quite different types of coloured bird species [37].

We found a negative correlation between land use richness (e.g., habitat diversity) and the degree of colour inequality in bird communities. This finding may be associated with the higher availability of niches [79], which increases the presence of more species, potentially of different colours. This association was also observed when considering potential habitat-related differences and when running the mixed-model procedure. The other metric related to landscape heterogeneity that was examined in this study, weighted edge density, exhibited a positive correlation with the colour inequality of avian communities. We can speculate that increasing the configurational heterogeneity of the landscapes attracts bird species typical of edge zones (e.g., *Muscicapa striata*, *Curruca nisoria* and *Sitta europaea*) [116], which exhibit more homogeneous colouration (e.g., predominantly grey and white). We found that an increase in species richness is associated with a decrease in the colouration inequality of the avian community. We interpret this result as evidence that, by increasing the overall number of species in a given community, the probability of increasing colour diversity (e.g., lower colour inequality) is higher by chance [45]. Finally, we found evidence that avian communities characterized by the presence of closely related species tend to exhibit a more diversified community colouration (e.g., lower colour inequality). This result supports previous studies indicating that closely related species coexisting in the same habitat often diverge to enhance species recognition [53,54].

## 5. Conclusions

Our findings constitute a large-scale spatial exploration (e.g., covering the entire Italian peninsula) of avian communities, using a quantitative approach to investigate the relative composition of birds’ colouration. This approach presents some limitations, mainly associated with the simplification of habitat characteristics used to explore changes in community colours. Here, we associated bird communities with their respective habitats. However, the complexity of some habitats makes it difficult to establish a direct correlation between the habitat characteristics and the birds’ colours. A better understanding of these associations could be achieved by incorporating information about habitat structure and ecological partitioning within the habitat, as well as obtaining effective measurements of the irradiance spectral signatures or background colours in different parts of those habitats [37]. For instance, we previously highlighted the differences within forest habitats based on the structure of understories (potentially hosting black, red, or brown-coloured species) and the canopy (potentially hosting blue, green, or yellow-coloured species) [15]. In consequence, we recognize that when working at a large spatial scale, it is necessary to accept some compromises, for instance, reducing the classification accuracy of habitats slightly but simultaneously increasing the overall sample size or spatial distribution of the data. Finally, we expect that further research efforts will incorporate plumage colouration and a more traditional trait-based approach to better capture the complexity of the association between communities, landscapes, and habitat characteristics.

## Figures and Tables

**Table 1:** Mean and standard deviation for species richness, phylogenetic relatedness (PSVs), and the mean percentage of colouration of breeding avian communities in different types of habitats in Italy. The avian composition at each of the 8508 sampling sites was determined by the presence of bird species during the breeding season. The values of the standard deviation for each percentage of colouration are presented in Table S3.

Habitat	SR (Mean)	SR (SD)	PSVs (Mean)	PSVs (SD)	Black % (Mean)	Blue % (Mean)	Brown % (Mean)	Green % (Mean)	Grey % (Mean)	Purple % (Mean)	Red % (Mean)	Rufous % (Mean)	White % (Mean)	Yellow % (Mean)
Coniferous forest	14.093	5.175	0.559	0.084	16.995	1.069	17.421	0.187	37.451	0.016	0.312	2.066	18.519	5.964
Deciduous forest	15.825	5.224	0.613	0.076	16.359	1.349	15.949	0.366	38.707	0.037	0.278	1.658	19.129	6.169
Shrubs	14.914	5.172	0.619	0.079	16.498	1.122	14.292	0.343	40.141	0.057	0.262	1.746	20.014	5.525
Non-agricultural herbaceous	15.662	5.892	0.579	0.096	17.357	0.989	14.854	0.293	38.112	0.067	0.360	2.103	20.060	5.804
Wetlands/Water	25.400	5.325	0.807	0.037	16.939	0.836	9.251	0.441	36.571	0.089	0.659	2.604	28.959	3.650
Bare soil and dunes	12.429	6.408	0.599	0.127	18.228	0.768	12.375	0.570	40.404	0.081	0.561	2.420	19.037	5.556
Orchards and vineyards	18.059	5.806	0.650	0.070	17.238	1.533	11.675	0.802	39.134	0.193	0.379	1.714	21.200	6.132
Forages	17.806	6.117	0.613	0.093	18.050	1.146	13.928	0.476	38.744	0.096	0.418	2.093	19.624	5.425
Croplands	19.11	6.045	0.700	0.080	17.132	1.047	12.794	0.674	38.911	0.171	0.403	2.246	20.992	5.631
Urban	16.837	4.795	0.667	0.076	18.934	1.266	9.489	0.498	42.318	0.185	0.379	1.542	20.861	4.530
Mixed	18.414	5.918	0.636	0.088	17.258	1.216	13.656	0.547	39.325	0.109	0.393	1.909	19.785	5.803
Total	17.744	5.954	0.649	0.092	17.207	1.188	13.542	0.541	39.151	0.121	0.372	1.959	20.194	5.724

**Table 2:** Results of generalized linear models accounting for a variation in the percentage of each different colour and colour inequality (e.g., Gini coefficient) in the breeding avian communities of Italy, regarding the type of habitat, landscape characteristics (e.g., configurational and compositional heterogeneity), the number of species in the community (i.e., species richness), phylogenetic relatedness (i.e., PSVs), latitude and longitude. The table reports only significant associations, and we used the sign of the estimates to determine the direction of the associations (e.g., positive or negative). The full outputs of every single model are reported in Table S5. N = 8508 sampled sites and 263 bird species.

Variable	Black %	Blue %	Brown %	Green %	Grey %	Purple %	Red %	Rufous %	White %	Yellow %	Colours Inequality
Coniferous forest	(−)	(+)	(+)	(−)	(−)	(−)	(−)	(−)			(−)
Deciduous forest	(−)	(+)	(+)	(−)	(−)		(−)	(−)		(+)	(−)
Shrubs		(+)	(+)	(−)			(−)	(−)			
Non-agricultural herbaceous			(+)	(−)	(−)		(−)				
Wetlands/Water			(−)	(−)	(−)				(+)	(−)	(+)
Bare soil and dunes											
Orchards and vineyards		(+)			(−)	(+)	(−)	(−)	(+)		
Forages		(+)	(+)		(−)		(−)	(−)			
Croplands		(+)			(−)	(+)	(−)		(+)		
Urban		(+)	(−)			(+)	(−)	(−)	(+)	(−)	(+)
Mixed		(+)	(+)		(−)		(−)	(−)			
LUR	(−)		(+)	(+)	(−)	(−)	(−)	(+)		(+)	(−)
WEDGE	(+)	(+)	(−)	(−)	(+)	(+)	(+)	(−)		(−)	(+)
Species richness	(−)	(−)	(+)	(+)	(−)	(−)	(+)	(+)		(+)	(−)
Phylogenetic relatedness—PSVs											(+)
Latitude	(+)	(−)	(+)	(−)	(−)	(+)	(+)	(+)	(−)		(−)
Longitude	(−)	(+)	(+)		(−)	(+)	(−)	(−)	(+)	(+)	(−)

## Data Availability

All relevant data are contained in the manuscript’s Supplementary Files. Data on bird species distribution in Italy are available upon request from Lipu Italy (Lipu—BirdLife Italy, 2012). The data about bird species colouration were published by Delhey et al. (2023) [15] and are publicly available at https://www.pnas.org/doi/10.1073/pnas.2217692120#supplementary-materials (accessed on 25 August 2024).

## Supplementary Materials

The following supporting information can be downloaded at https://www.mdpi.com/article/10.3390/ani16050815/s1. Table S1: Results of Mantel test; Table S2: List of 262 bird species recorded during the breeding season in Italy, with the number of observations and relative percentage from the total of sampling sites; Table S3: Standard deviation (SD) of the percentage of colouration of breeding avian communities in different types of environments in Italy; Table S4: Description of the inequality of colour composition in breeding avian communities of Italy; Table S5: Full outputs of generalized linear models accounting for variation in the percentage of each different colour and colour inequality in the breeding avian communities of Italy, regarding the type of habitat, landscape characteristics, the number of species in the community and latitude and longitude; Table S6: Result of the generalized linear mixed model, accounting for variation in the colour inequality in the breeding avian communities of Italy, regarding landscape characteristics, the number of species in the community, phylogenetic relatedness, latitude and longitude; Figure S1: The sum of different colours in the breeding avian communities by type of habitat; and Figure S2: Fan dendrogram representation of the 262 bird species recorded during the breeding season in Italy.

## Abbreviations

The following abbreviations are used in this manuscript:

PSV	Phylogenetic relatedness
MCC	Maximum clade credibility tree
LUR	Land use richness
WEDGE	Weighted edge density
SAC	spatial autocorrelation
GLM	Generalized linear model
GLMM	Generalized linear mixed model
UHI	Urban heat island
