# Supplementary material for: Bird Community Colours Across Different Types of Habitat
Source: Animals (Basel). 2026 Mar 5;16(5):815. doi: 10.3390/ani16050815 (PMC12985330; doi:10.3390/ani16050815)

Supplementary Material - Bird community colours across different types of habitat

Table S1. Results of Mantel test (Mantel statistic - r_M and p values) for spatial autocorrelation, obtained after 999 permutations for the percentage of each different colour (e.g., black, blue, brown, green, grey, purple, red, rufous, white and yellow) and colour inequality (e.g., Gini coefficient) in the breeding avian communities of Italy.

**Variables**	**r_M**	**p-value**
Black %	0.047	0.010
Blue %	0.084	0.010
Brown %	0.010	0.160
Green %	-0.007	0.690
Grey %	0.013	0.170
Purple %	0.066	0.011
Red %	-0.012	0.880
Rufous %	0.005	0.350
White %	-0.010	0.560
Yellow %	-0.004	0.620
Colours inequality	-0.002	0.520

Table S2. List of 262 bird species recorded during the breeding season in Italy, with the number of observations and relative percentage from the total of sampling sites (8,508). The species are ordered by commonness (e.g., from high to low observation percentage).

Species	Observations	Percentage
Turdus merula	6600	77.57
Sylvia atricapilla	6534	76.80
Parus major	5154	60.58
Corvus cornix	5032	59.14
Columba palumbus	4939	58.05
Fringilla coelebs	4319	50.76
Pica pica	3902	45.86
Hirundo rustica	3779	44.42
Streptopelia decaocto	3704	43.54
Carduelis carduelis	3681	43.27
Apus apus	3580	42.08
Serinus serinus	3540	41.61
Passer italiae	3488	41.00
Luscinia megarhynchos	3097	36.40
Cyanistes caeruleus	2941	34.57
Garrulus glandarius	2858	33.59
Erithacus rubecula	2776	32.63
Sturnus vulgaris	2677	31.46
Cuculus canorus	2478	29.13
Troglodytes troglodytes	2448	28.77
Emberiza cirlus	2411	28.34
Streptopelia turtur	2332	27.41
Columba livia livia	2213	26.01
Chloris chloris	2173	25.54
Curruca melanocephala	2049	24.08
Oriolus oriolus	1957	23.00
Picus viridis	1919	22.56
Dendrocopos major	1886	22.17
Delichon urbicum	1818	21.37
Passer montanus	1781	20.93
Emberiza calandra	1756	20.64
Buteo buteo	1669	19.62
Falco tinnunculus	1603	18.84
Phylloscopus collybita	1591	18.70
Cisticola juncidis	1543	18.14
Phoenicurus phoenicurus	1422	16.71
Merops apiaster	1314	15.44
Galerida cristata	1272	14.95
Larus michahellis	1247	14.66

Curruca cantillans	1237	14.54
Periparus ater	1186	13.94
Upupa epops	1181	13.88
Turdus philomelos	1171	13.76
Motacilla alba	1169	13.74
Certhia brachydactyla	1159	13.62
Phasianus colchicus	1126	13.23
Cettia cetti	1034	12.15
Linaria cannabina	938	11.02
Lullula arborea	930	10.93
Alauda arvensis	923	10.85
Corvus monedula	903	10.61
Passer hispaniolensis	902	10.60
Ardea cinerea	870	10.23
Aegithalos caudatus	840	9.87
Sitta europaea	828	9.73
Regulus ignicapillus	809	9.51
Sturnus unicolor	793	9.32
Turdus viscivorus	787	9.25
Phoenicurus ochruros	761	8.94
Coturnix coturnix	691	8.12
Hippolais polyglotta	680	7.99
Curruca communis	662	7.78
Muscicapa striata	611	7.18
Saxicola rubicola	608	7.15
Ardea ibis	607	7.13
Poecile palustris	596	7.01
Anas platyrhynchos	582	6.84
Motacilla flava	566	6.65
Egretta garzetta	564	6.63
Rhadina bonelli	460	5.41
Lanius collurio	443	5.21
Corvus corax	442	5.20
Gallinula chloropus	425	5.00
Motacilla cinerea	411	4.83
Anthus trivialis	355	4.17
Phalacrocorax carbo	353	4.15
Corvus corone	341	4.01
Ptyonoprogne rupestris	337	3.96
Milvus migrans	316	3.71
Regulus regulus	288	3.39
Pernis apivorus	280	3.29
Nycticorax nycticorax	264	3.10
Pyrrhula pyrrhula	256	3.01
Lophophanes cristatus	250	2.94
Jynx torquilla	224	2.63

Poecile montanus	217	2.55
Vanellus vanellus	214	2.52
Emberiza cia	209	2.46
Calandrella brachydactyla	208	2.44
Athene noctua	204	2.40
Certhia familiaris	201	2.36
Prunella modularis	193	2.27
Nucifraga caryocatactes	185	2.17
Acrocephalus scirpaceus	184	2.16
Accipiter nisus	179	2.10
Loxia curvirostra	174	2.05
Falco subbuteo	163	1.92
Burhinus oedicnemus	162	1.90
Oenanthe oenanthe	159	1.87
Emberiza citrinella	159	1.87
Anthus campestris	155	1.82
Lanius senator	154	1.81
Dryocopus martius	151	1.77
Anthus spinoletta	145	1.70
Ardea purpurea	143	1.68
Acrocephalus palustris	142	1.67
Acrocephalus arundinaceus	138	1.62
Fulica atra	136	1.60
Melanocorypha calandra	135	1.59
Threskiornis aethiopicus	129	1.52
Milvus milvus	127	1.49
Circus aeruginosus	122	1.43
Ardea alba	119	1.40
Curruca curruca	119	1.40
Apus pallidus	115	1.35
Falco naumanni	105	1.23
Tachymarptis melba	100	1.18
Coracias garrulus	99	1.16
Microcarbo pygmaeus	98	1.15
Turdus pilaris	98	1.15
Saxicola rubetra	90	1.06
Circaetus gallicus	87	1.02
Falco peregrinus	84	0.99
Curruca conspicillata	83	0.98
Chroicocephalus ridibundus	82	0.96
Dryobates minor	82	0.96
Carduelis spinus	79	0.93
Emberiza hortulana	79	0.93
Carduelis flammea	75	0.88
Monticola solitarius	74	0.87
Leiothrix lutea	74	0.87

Coccothraustes coccothraustes	74	0.87
Alcedo atthis	73	0.86
Turdus torquatus	72	0.85
Himantopus himantopus	70	0.82
Sylvia borin	64	0.75
Sterna hirundo	62	0.73
Rhadina sibilatrix	62	0.73
Aquila chrysaetos	61	0.72
Pyrrhocorax graculus	61	0.72
Tachybaptus ruficollis	59	0.69
Podiceps cristatus	58	0.68
Cinclus cinclus	53	0.62
Alectoris barbara	47	0.55
Ichthyaetus melanocephalus	46	0.54
Cygnus olor	45	0.53
Passer domesticus	44	0.52
Petronia petronia	44	0.52
Tadorna tadorna	43	0.51
Ciconia ciconia	42	0.49
Ardeola ralloides	38	0.45
Charadrius dubius	37	0.43
Circus pygargus	33	0.39
Psittacula krameri	31	0.36
Otus scops	31	0.36
Alectoris rufa	30	0.35
Curruca undata	30	0.35
Astur gentilis	28	0.33
Falco vespertinus	28	0.33
Strix aluco	27	0.32
Monticola saxatilis	25	0.29
Pyrrhocorax pyrrhocorax	25	0.29
Gelochelidon nilotica	24	0.28
Prunella collaris	23	0.27
Gyps fulvus	22	0.26
Caprimulgus europaeus	22	0.26
Phoenicopterus roseus	21	0.25
Colinus virginianus	20	0.24
Lanius minor	19	0.22
Haematopus ostralegus	17	0.20
Picus canus	17	0.20
Ixobrychus minutus	16	0.19
Oenanthe hispanica	16	0.19
Carduelis citrinella	16	0.19
Plegadis falcinellus	15	0.18
Tringa ochropus	14	0.16
Columba oenas	14	0.16

Curruca sarda	14	0.16
Remiz pendulinus	14	0.16
Tetrao tetrix	13	0.15
Riparia riparia	13	0.15
Anser anser	12	0.14
Alectoris graeca	12	0.14
Gypaetus barbatus	11	0.13
Actitis hypoleucos	11	0.13
Ficedula albicollis	11	0.13
Emberiza melanocephala	11	0.13
Tringa totanus	10	0.12
Sturnus roseus	10	0.12
Myiopsitta monachus	10	0.12
Numenius arquata	9	0.11
Sternula albifrons	9	0.11
Platalea leucorodia	8	0.09
Mergus merganser	8	0.09
Montifringilla nivalis	8	0.09
Ciconia nigra	7	0.08
Chroicocephalus genei	7	0.08
Anthus pratensis	7	0.08
Phalacrocorax aristotelis	6	0.07
Botaurus stellaris	6	0.07
Falco biarmicus	6	0.07
Rallus aquaticus	6	0.07
Aythya ferina	5	0.06
Falco eleonorae	5	0.06
Recurvirostra avosetta	5	0.06
Tringa nebularia	5	0.06
Picoides tridactylus	5	0.06
Phylloscopus trochilus	5	0.06
Spatula clypeata	4	0.05
Aythya fuligula	4	0.05
Tetrax tetrax	4	0.05
Tyto alba	4	0.05
Dendrocoptes medius	4	0.05
Carduelis corsicana	4	0.05
Emberiza fucata	3	0.04
Tetrao urogallus	3	0.04
Perdix perdix	3	0.04
Crex crex	3	0.04
Charadrius alexandrinus	3	0.04
Limosa limosa	3	0.04
Tringa glareola	3	0.04
Hydrocoloeus minutus	3	0.04
Ichthyaetus audouinii	3	0.04

Chlidonias niger	3	0.04
Chlidonias leucopterus	3	0.04
Cecropis daurica	3	0.04
Podiceps nigricollis	2	0.02
Cairina moschata	2	0.02
Netta rufina	2	0.02
Aythya nyroca	2	0.02
Hieraaetus pennatus	2	0.02
Lagopus mutus	2	0.02
Francolinus francolinus	2	0.02
Glareola pratincola	2	0.02
Tringa erythropus	2	0.02
Thalasseus sandvicensis	2	0.02
Chlidonias hybrida	2	0.02
Hippolais icterina	2	0.02
Ficedula hypoleuca	2	0.02
Calonectris diomedea	1	0.01
Mareca strepera	1	0.01
Anas crecca	1	0.01
Spatula querquedula	1	0.01
Neophron percnopterus	1	0.01
Aquila fasciata	1	0.01
Falco columbarius	1	0.01
Porzana porzana	1	0.01
Charadrius hiaticula	1	0.01
Pluvialis squatarola	1	0.01
Calidris alpina	1	0.01
Lymnocryptes minimus	1	0.01
Gallinago gallinago	1	0.01
Arenaria interpres	1	0.01
Larus cachinnans	1	0.01
Hydroprogne caspia	1	0.01
Clamator glandarius	1	0.01
Asio otus	1	0.01
Dendrocopos leucotos	1	0.01
Locustella luscinioides	1	0.01
Acrocephalus schoenobaenus	1	0.01
Iduna pallida	1	0.01
Tichodroma muraria	1	0.01

Table S3. Standard deviation (SD) of the percentage of colouration of breeding avian communities in different types of environments in Italy. The avian composition in each of the 8,508 sampling sites was based on the presence of 262 bird species during the breeding season.

Environment	Black % (SD)	Blue % (SD)	Brown % (SD)	Green % (SD)	Grey % (SD)	Purple % (SD)	Red % (SD)	Rufous % (SD)	White % (SD)	Yellow % (SD)	(n)
Conifers forest	3.000	0.685	4.208	0.424	3.816	0.045	0.278	0.913	2.621	1.534	480
Deciduous forest	2.569	0.674	3.316	0.584	3.344	0.071	0.312	0.670	2.253	1.435	1275
Shrubs	2.864	0.841	3.607	0.709	3.586	0.100	0.278	0.868	3.245	1.581	106
Non-agricultural herbaceous	3.063	0.719	3.664	0.554	3.922	0.098	0.259	0.907	3.473	1.414	237
Wetlands/Water	2.761	0.794	3.382	0.476	4.407	0.090	0.336	0.891	7.283	1.388	20
Bare soil and dunes	3.464	0.708	3.842	0.895	3.651	0.111	0.261	0.641	2.837	1.609	28
Orchards and vineyards	2.382	0.730	2.890	0.827	3.380	0.117	0.334	0.750	3.334	1.401	595
Forages	3.070	0.660	3.939	0.605	3.767	0.119	0.318	0.897	2.886	1.551	268
Croplands	2.979	0.606	3.488	0.690	3.551	0.126	0.279	0.809	3.609	1.642	2673
Urban	2.664	0.711	2.884	0.800	3.650	0.130	0.342	0.778	3.312	1.505	603
Mixed	2.735	0.683	3.700	0.705	3.485	0.122	0.319	0.817	3.117	1.579	2223

Table S4. Description of the inequality of colour composition (e.g., Gini coefficient) in breeding avian communities of Italy, based on the presence of 262 bird species in different types of habitat. The values shown are the mean, minimum, maximum, and number of observations (n).

Habitat	Colours inequality (mean)	Colours inequality (min)	Colours inequality (max)	Colours inequality (n)
Conifers forest	0.682	0.609	0.822	480
Deciduous forest	0.684	0.580	0.801	1275
Shrubs	0.699	0.619	0.781	106
Non-agricultural herbaceous	0.685	0.603	0.761	237
Wetlands/Water	0.712	0.657	0.774	20
Bare soil and dunes	0.695	0.620	0.770	28
Orchards and vineyards	0.684	0.574	0.797	595
Forages	0.687	0.591	0.777	268
Croplands	0.687	0.584	0.811	2673
Urban	0.718	0.617	0.824	603
Mixed	0.687	0.579	0.816	2223

Table S5. Full outputs of generalized linear models accounting for variation in the percentage of each different colour (e.g., black, blue, brown, green, grey, purple, red, rufous, white and yellow) and colour inequality (e.g., Gini coefficient) in the breeding avian communities of Italy, regarding the type of habitat, landscape characteristics (e.g., configurational and compositional heterogeneity), the number of species in the community (e.g., species richness) and latitude and longitude. The table reports only significant associations and provides the goodness of fit of each model (R^2). Note that the values of the estimate not shown in the table correspond to the type of habitat used for the comparison with all the other habitat categories. N = 8,508 sampled sites and 262 bird species.

Variable	Estimate	Std. Error	t value	p-value
**Model: Black percentage (R^2 = 0.14)**				
Habitat (Coniferous forest)	-1.429	0.516	-2.771	0.006
Habitat (Deciduous forest)	-1.540	0.508	-3.033	0.002
LUR	-0.188	0.039	-4.804	< 2e-16
WEDGE	2.996	0.373	8.028	< 2e-16
Species richness	-0.055	0.005	-11.165	< 2e-16
Latitude	0.186	0.013	14.185	< 2e-16
Longitude	-0.163	0.013	-12.979	< 2e-16
**Model: Blue percentage (R^2 = 0.11)**				
Habitat (Coniferous forest)	0.341	0.127	2.680	0.007
Habitat (Croplands)	0.292	0.125	2.340	0.019
Habitat (Deciduous forest)	0.577	0.125	4.602	4e-06
Habitat (Forages)	0.441	0.130	3.377	0.001
Habitat (Mixed)	0.416	0.126	3.316	0.001
Habitat (Orchards and vineyards)	0.655	0.127	5.143	3e-07
Habitat (Shrubs)	0.347	0.140	2.473	0.013
Habitat (Urban)	0.456	0.128	3.574	0.000
WEDGE	0.202	0.092	2.191	0.029
Species richness	-0.007	0.001	-5.293	1e-07
Latitude	-0.013	0.003	-4.151	3e-05
Longitude	0.036	0.003	11.725	< 2e-16
**Model: Brown percentage (R^2 = 0.24)**				
Habitat (Coniferous forest)	5.204	0.672	7.746	1e-14
Habitat (Deciduous forest)	3.899	0.661	5.895	4e-09
Habitat (Forages)	1.976	0.688	2.870	0.004
Habitat (Mixed)	2.026	0.662	3.058	0.002
Habitat (Non-agricultural herbaceous)	2.681	0.691	3.878	1e-04
Habitat (Shrubs)	2.388	0.739	3.229	0.001
Habitat (Urban)	-1.794	0.674	-2.663	0.008
Habitat (Wetlands/Water)	-3.264	1.015	-3.216	0.001

LUR	0.450	0.051	8.808	< 2e-16
WEDGE	-6.257	0.486	-12.869	< 2e-16
Species richness	0.021	0.007	3.255	0.001
Latitude	0.078	0.017	4.559	5e-06
Longitude	0.153	0.016	9.329	< 2e-16
**Model: Green percentage (R^2 = 0.08)**				
Habitat (Coniferous forest)	-0.410	0.130	-3.147	0.002
Habitat (Deciduous forest)	-0.287	0.128	-2.234	0.026
Habitat (Non-agricultural herbaceous)	-0.353	0.134	-2.63	0.009
Habitat (Shrubs)	-0.318	0.143	-2.213	0.027
Habitat (Wetlands/Water)	-0.391	0.197	-1.983	0.047
LUR	0.039	0.010	3.893	1e-04
WEDGE	-0.233	0.094	-2.474	0.013
Species richness	0.018	0.001	14.907	< 2e-16
Latitude	-0.008	0.003	-2.405	0.016
**Model: Grey percentage (R^2 = 0.11)**				
Environment (Coniferous forest)	-3.073	0.673	-4.566	5e-06
Environment (Croplands)	-1.602	0.660	-2.429	0.015
Environment (Deciduous forest)	-2.032	0.663	-3.066	0.002
Environment (Forages)	-1.989	0.690	-2.884	0.004
Environment (Mixed)	-1.758	0.663	-2.649	0.008
Environment (Non-agricultural herbaceous)	-2.496	0.692	-3.604	0.000
Environment (Orchards and vineyards)	-1.456	0.673	-2.163	0.031
Environment (Wetlands/Water)	-3.340	1.017	-3.285	0.001
LUR	-0.373	0.051	-7.302	3.1e-13
WEDGE	5.764	0.487	11.836	< 2e-16
Species richness	-0.056	0.007	-8.467	< 2e-16
Latitude	-0.134	0.017	-7.796	7.1e-15
Longitude	-0.194	0.016	-11.811	< 2e-16
**Model: Purple percentage (R^2 = 0.25)**				
Environment (Coniferous forest)	-0.055	0.021	-2.586	0.010
Environment (Croplands)	0.102	0.021	4.832	1e-06
Environment (Orchards and vineyards)	0.105	0.021	4.882	1e-06
Environment (Urban)	0.105	0.021	4.887	1e-06
LUR	-0.004	0.002	-2.526	0.012
WEDGE	0.057	0.016	3.667	0.000
Species richness	-0.001	0.000	-6.970	3.1e-12
Latitude	0.002	0.001	2.761	0.006
Longitude	0.010	0.001	18.431	< 2e-16
**Model: Red percentage (R^2 = 0.09)**				
Environment (Coniferous forest)	-0.280	0.057	-4.879	1.1e-06
Environment (Croplands)	-0.194	0.056	-3.459	0.001
Environment (Deciduous forest)	-0.290	0.056	-5.132	2.9e-07
Environment (Forages)	-0.207	0.059	-3.520	4.3e-04
Environment (Mixed)	-0.209	0.057	-3.694	2.2e-04
Environment (Non-agricultural herbaceous)	-0.204	0.059	-3.453	0.001

Environment (Orchards and vineyards)	-0.164	0.057	-2.868	0.004
Environment (Shrubs)	-0.250	0.063	-3.968	7.3e-05
Environment (Urban)	-0.219	0.057	-3.811	1.4e-04
LUR	-0.012	0.004	-2.659	0.008
WEDGE	0.142	0.041	3.423	0.001
Species richness	0.010	0.001	17.064	< 2e-16
Latitude	0.018	0.001	12.569	< 2e-16
Longitude	-0.004	0.001	-2.932	0.003
**Model: Rufous percentage (R^2 = 0.16)**				
Environment (Coniferous forest)	-0.405	0.149	-2.719	0.007
Environment (Deciduous forest)	-0.702	0.147	-4.782	1.7e-06
Environment (Forages)	-0.391	0.153	-2.559	0.011
Environment (Mixed)	-0.384	0.147	-2.613	0.009
Environment (Orchards and vineyards)	-0.507	0.149	-3.401	0.001
Environment (Shrubs)	-0.512	0.164	-3.118	0.002
Environment (Urban)	-0.697	0.150	-4.659	3.2e-06
LUR	0.035	0.011	3.133	0.002
WEDGE	-0.950	0.108	-8.802	< 2e-16
Species richness	0.021	0.001	14.574	< 2e-16
Latitude	0.051	0.004	13.550	< 2e-16
Longitude	-0.021	0.004	-5.817	6.2e-09
**Model: White percentage (R^2 = 0.11)**				
Environment (Croplands)	1.598	0.600	2.661	0.008
Environment (Orchards and vineyards)	1.461	0.612	2.386	0.017
Environment (Urban)	1.753	0.614	2.856	0.004
Environment (Wetlands/Water)	9.650	0.925	10.430	<2e-16
Latitude	-0.180	0.016	-11.522	<2e-16
Longitude	0.037	0.015	2.46	0.014
**Model: Yellow percentage (R^2 = 0.14)**				
Environment (Deciduous forest)	0.560	0.285	1.967	0.049
Environment (Urban)	-1.026	0.290	-3.535	0.000
Environment (Wetlands/Water)	-2.354	0.437	-5.384	7.5e-08
LUR	0.096	0.022	4.350	1.4e-05
WEDGE	-1.019	0.209	-4.867	1.1e-06
Species richness	0.038	0.003	13.340	< 2e-16
Longitude	0.140	0.007	19.926	< 2e-16
**Model: Colours inequality (R^2 = 0.17)**				
Environment (Deciduous forest)	-0.011	0.005	-2.112	0.035
Environment (Urban)	0.016	0.005	2.922	0.003
Environment (Wetlands/Water)	0.020	0.008	2.531	0.011
LUR	-0.003	0.000	-8.461	< 2e-16
WEDGE	0.045	0.004	11.764	< 2e-16
Species richness	-0.002	0.000	-30.199	< 2e-16
PSVs	0.078	0.004	20.661	< 2e-16
Latitude	-0.001	0.000	-8.741	< 2e-16
Longitude	-0.002	0.000	-14.456	< 2e-16

Table S6. Result of the generalized linear mixed model, accounting for variation in the colour inequality (e.g., Gini coefficient) in the breeding avian communities of Italy, regarding landscape characteristics (e.g., configurational and compositional heterogeneity), the number of species in the community (i.e., species richness), phylogenetic relatedness (i.e., PSVs), latitude and longitude. The modelling procedure incorporated the type of habitat as a random factor (groups = 11). The table reports only significant associations. N = 8,508 sampled sites and 262 bird species. Model fit: Pseudo-R2 (fixed effects) = 0.14, Pseudo-R2 (total) = 0.23. Random effects: Type of habitat Std. Dev. = 0.009; residual Std. Dev. = 0.027.

**Variable**	**Estimate**	**Std. Error**	**t value**	**p-value**
LUR	-0.003	4e-4	-8.516	< 2e-16
WEDGE	0.046	0.004	11.851	< 2e-16
Species richness	-0.002	5e-4	-30.185	< 2e-16
PSVs	0.078	0.004	20.846	< 2e-16
Latitude	-0.001	1.4e-4	-8.754	< 2e-16
Longitude	-0.001	1.3e-4	-14.468	< 2e-16

Figure S1. The sum of different colours (e.g., black (A), blue (B), brown (C), green (D), grey (E), purple (F), red (G), rufous (H), white (I) and yellow (J) in the breeding avian communities by type of habitat. Box plots show the median (the black bar in the middle of rectangles), upper and lower quartiles (length of rectangles), maximum and minimum values (whiskers), and mean values (red dots). N = 8,508.

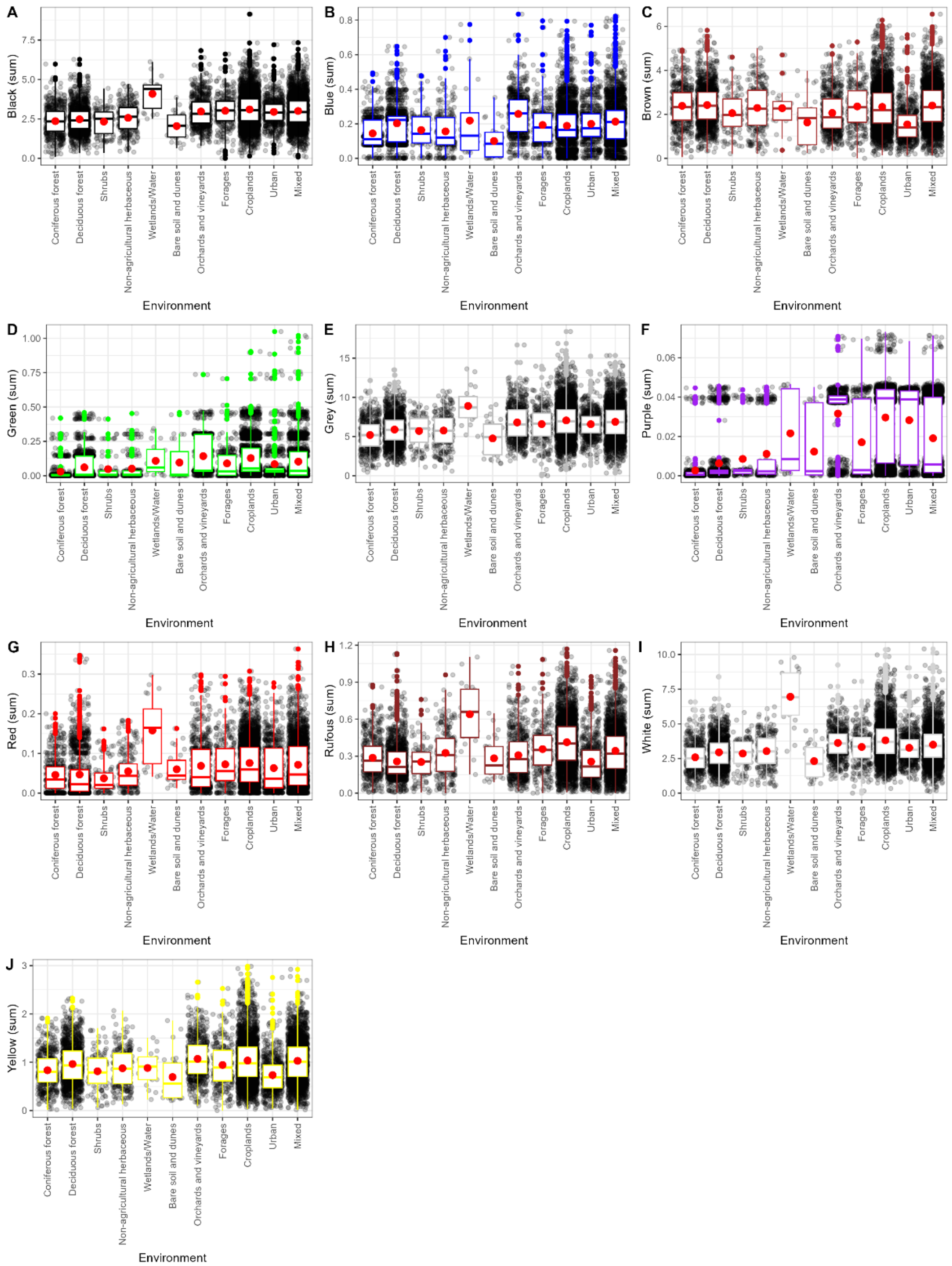

Figure S2. Fan dendrogram representation of the 262 bird species recorded during the breeding season in Italy, in 8,508 sampling sites. The figure is based on the Maximum Clade Credibility tree (MCC) obtained from 100 species-level phylogenies downloaded from the BirdTree web tool (http://birdtree.org) (Jetz et al., 2012).

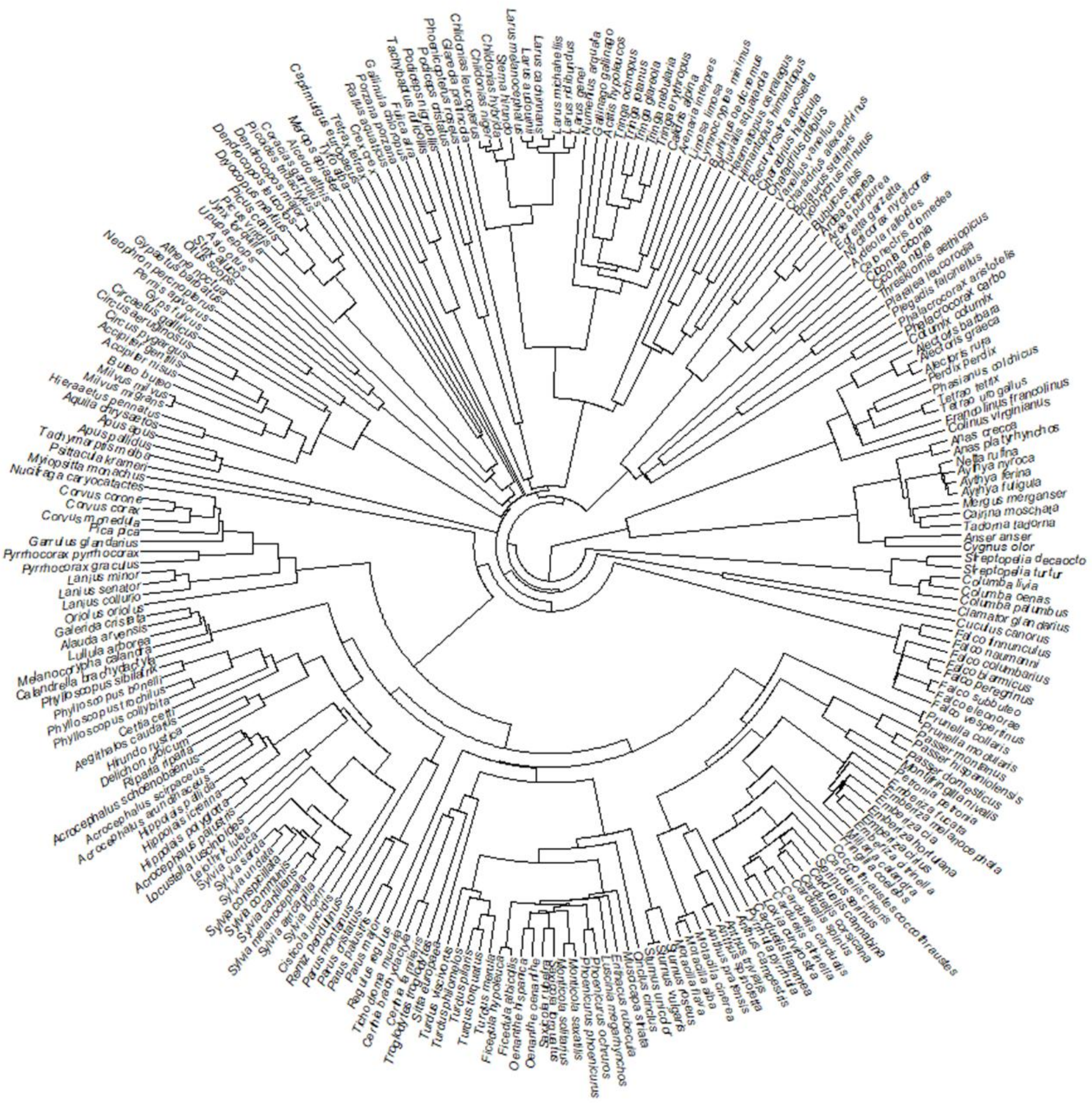

Supplement: Supplementary file 1 [file animals-16-00815-s001.zip › animals-4151304-supplementary.pdf]
